# Availability and Functionality of Physical Health and Resuscitation Equipment in an Inpatient Setting: A Closed Loop Audit Cycle

**DOI:** 10.1192/bjo.2022.496

**Published:** 2022-06-20

**Authors:** Song Ling Tang, Ikhlas Fadlalla

**Affiliations:** 1 Norfolk and Norwich University Hospitals NHS Foundation Trust, Norwich, United Kingdom; 2 Norfolk and Suffolk NHS Foundation Trust, Norwich, United Kingdom

## Abstract

**Aims:**

To ensure physical health and resuscitation equipment on all wards in a mental health hospital fulfil relevant standards. A closed-loop audit of this was performed on four acute adult inpatient wards after implementing interventions.

**Methods:**

Data were collected from treatment rooms on each ward, with standards based on Physical Health in Mental Health; Final Report of a Scoping Group (Royal College of Psychiatrists) and Mental Health Inpatient Care Equipment and Drug Lists (Resuscitation Council UK) – parallel to the trust approved standards.

Percentage of availability and functionality against audit standards were tabulated and interventions were carried out, including:

1. Awareness presentations at trust clinical governance meetings.
2. Each ward to have own complete sets of physical health and resuscitation equipment.
3. Policy for wards to register their physical health equipment and service details on the trust maintenance services database. The medical device engineering team to complete maintenance and repair as needed.
4. Resuscitation equipment on each ward being checked weekly and replaced as needed (monthly before).

A re-audit was performed one year post intervention on four acute adult inpatient wards in the mental health hospital using similar parameters.

**Results:**

1. In general, 90.0% of the standards are met (out of 160 pieces of equipment, 144 are in stock and functional), similar to that of previous year (90.0%).
2. Decrease in overall available and functional physical health equipment: 76.6% (49/64) compared to 83.8% last year.
3. Increase in overall in overall available and functional resuscitation equipment: 99.2% (95/96) compared to 94.2% last year.

**Conclusion:**

There is a significant decrease in percentage of overall available and functional physical health equipment; while that of resuscitation equipment has significantly improved when checked and corrected weekly using the trust Resuscitation Check Form.

Action plan:

1. All unavailable/ inadequate equipment to be reordered or sent for maintenance immediately.
2. Discussion in the upcoming trust Physical Health Nurses Forum and Medical Devices Standards Group on audit recommendations below:
  - . Allocation of named permanent staff member to check presence and functionality of medical equipment regularly.
  - *a.* Creating a checklist similar to the Resuscitation Check Form for physical health equipment.
3. Discussion in the trust Resus Standards Group on ‘My Kit Check’ (MKC), a centrally monitored electronic checking platform with alerts automatically sent for incomplete checks or expired resuscitation items (e.g., AED batteries, anaphylaxis kit) that are not replaced. A funding request has been submitted for this.

